# Fabrication of wafer-scale nanoporous AlGaN-based deep ultraviolet distributed Bragg reflectors via one-step selective wet etching

**DOI:** 10.1038/s41598-022-25712-2

**Published:** 2022-12-27

**Authors:** Yongming Zhao, Maocheng Shan, Zhihua Zheng, Pengcheng Jian, WeiJie Liu, Shizhou Tan, Changqing Chen, Feng Wu, Jiangnan Dai

**Affiliations:** Wuhan National Laboratory for Optoelectronics, Huazhong University of Science and Technology, Wuhan, 430074 People’s Republic of China

**Keywords:** Chemistry, Materials science, Nanoscience and technology, Optics and photonics

## Abstract

In this paper, we reported on wafer-scale nanoporous (NP) AlGaN-based deep ultraviolet (DUV) distributed Bragg reflectors (DBRs) with 95% reflectivity at 280 nm, using epitaxial periodically stacked n-Al_0.62_Ga_0.38_N/u-Al_0.62_Ga_0.38_N structures grown on AlN/sapphire templates via metal–organic chemical vapor deposition (MOCVD). The DBRs were fabricated by a simple one-step selective wet etching in heated KOH aqueous solution. To study the influence of the temperature of KOH electrolyte on the nanopores formation, the amount of charge consumed during etching process was counted, and the surface and cross-sectional morphology of DBRs were characterized by Scanning electron microscopy (SEM) and atomic force microscopy (AFM). As the electrolyte temperature increased, the nanopores became larger while the amount of charge reduced, which revealed that the etching process was a combination of electrochemical and chemical etching. The triangular nanopores and hexagonal pits further confirmed the chemical etching processes. Our work demonstrated a simple wet etching to fabricate high reflective DBRs, which would be useful for AlGaN based DUV devices with microcavity structures.

## Introduction

AlGaN-based deep ultraviolet (DUV) vertical-cavity surface-emitting laser, resonant cavity LEDs and detectors have broad potential applications in sterilization, communication, data storage, biochemical detection. High reflectivity nitride distributed Bragg reflectors (DBRs), consisting of alternating layers with high and low refractive index, are important requirements for the operation of these devices with microcavity structures^1^. During the past decades, only a few groups have reported on the preparation of nitride deep ultraviolet (DUV) DBRs, mainly AlN/AlGaN and AlInN/AlGaN^2–8^. Unfortunately, they exhibited inadequate reflectivity due to low refractive index contrast, or limited crystal quality due to large lattice and thermal mismatch.

In recent years, electrochemical etching (ECE) has become a feasible method for the preparation of nitride DBRs which consist of periodically stacked n-doped AlGaN and undoped AlGaN^9,10^. As the n-doped AlGaN is selectively etched into porous morphology, a large refractive index contrast is formed between n-doped and undoped layers, and same Al content avoids lattice and thermal mismatch^11^. At present, laterally ECE and vertically-laterally ECE are the two main methods for preparing nanoporous (NP)-DBRs^12,13^. Using these two methods, GaN-based NP-DBRs have been widely reported in the blue and near-ultraviolet spectral regions^14–17^. NP-DBRs with high reflectivity (> 95%) were achieved and have been successfully coupled into LEDs, detectors, VCSELs and other devices, which demonstrated the viability of the NP-DBRs^10,18–20^. However, the complex processes such as photolithography and inductively coupled plasma (ICP) etching are required for laterally ECE to expose the sidewalls. Moreover, the small area of DBRs limits their viability for practical optoelectronic devices on a large scale. Compared with laterally ECE, vertically-laterally ECE exploits a simple one-step selective etching and can prepare wafer-scale DBRs^1,19–21^. However, few studies about the DUV NP-DBRs fabricated by the vertically-laterally have been reported. To meet the urgent needs of microcavity structures for DUV devices, large scale and high reflectivity DUV NP-DBRs should be developed.

In this work, we have fabricated wafer-scale DUV NP-DBRs with a high reflectivity (> 95%) by the simple one-step vertically-laterally ECE in heated KOH aqueous solution. The influence of electrolyte temperature on the porosity of the n-AlGaN layer was studied in detail, and the formation mechanisms of nanopores under the synergic actions of ECE and chemical etching (CE) was clarified.

## Experimental

**Figure 1:**
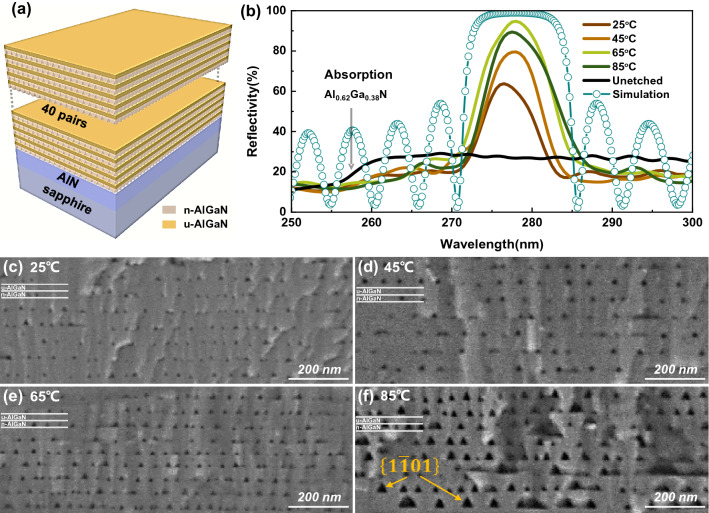
(**a**) Schematic structure; (**b**) The reflectivity spectra of DBRs prepared at 25 V DC bias in KOH electrolytes at different electrolyte temperatures, unetched sample and simulation at 65 °C; Cross-sectional SEM images of the DBRs etched at 25 V in KOH solution at (**c**) 25 °C, (**d**) 45 °C, (**e**) 65 °C and (**f**) 85 °C.

As shown in Fig. 1a, the epitaxial structure consisted of a 1.5 μm-thick AlN buffer and 40 pairs of n-Al_0.62_Ga_0.38_N/unintentional Al_0.62_Ga_0.38_N (28 nm/30 nm) DBRs layers, which were grown on 2-in. *c*-plane sapphire substrates by metal–organic chemical vapor deposition (MOCVD). During the growth, trimethylaluminum (TMA), trimethylgallium (TMG) and ammonia (NH_3_) were used as Al, Ga and N sources, respectively. Silane (SiH_4_) was the n-type dopant gases. Hydrogen was used as the carrier gas. The surface of AlGaN films was III-polar in this growth mode. The silicon doping concentration of n-Al_0.62_Ga_0.38_N was 4 × 10^18^ cm^−2^, and unintentional Al_0.62_Ga_0.38_N (u-Al_0.62_Ga_0.38_N) was about 1 × 10^16^ cm^−2^. Before electrochemical etching, the good electrical contact was formed by soldering indium at the sample edge. Then the AlGaN samples were anodization etched for 5 min, in 1 M KOH aqueous solution, with a platinum (Pt) plate as the counter electrode. The KOH aqueous solution was heated by an electric heating panel for constant temperature during etching. After etching, the samples were rinsed in deionized water and dried in N_2_. Scanning electron microscopy (SEM, Gemini300) and atomic force microscopy (AFM, SPM9700) were used to characterize the surface and cross-sectional morphologies of the samples. The reflectivity was measured with a UV–Vis spectrophotometer (SolidSpec-3700). The standard reference mirror (R > 99%, 200–400 nm) was used in the reflectivity spectra measurement. The finite element simulation of reflectivity was done using the Comsol Multiphysics software. To simplify the simulation, a bulk material with the same effective refractive index replaces the nanoporous layer. Keithley 2400C source meter provides DC constant bias and real-time monitoring and recording current through software.

## Results and discussion

**Figure 2:**
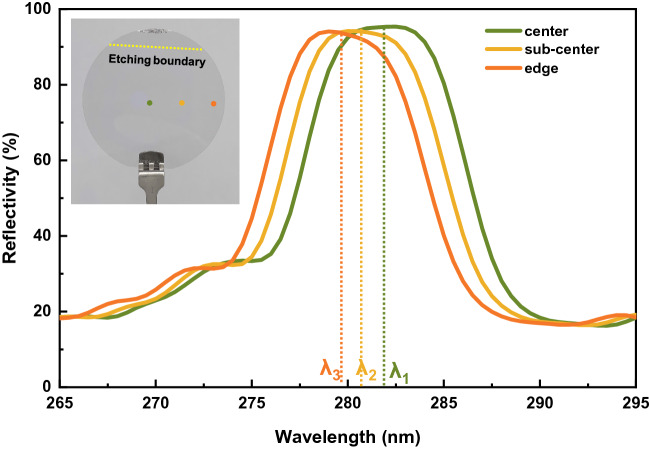
Reflectivity spectra of the 2-in. DBR wafer prepared at 25 V and 65 °C, the inset shows the photo of the DBR.

Figure 1B shows the reflectivity spectra of NP-DBRs etched at 25 V DC bias at different electrolyte temperatures. The reflectivity of the unetched sample was low in the DUV region (< 30%), and significantly declined at 257 nm corresponding to the absorption edge of Al_0.62_Ga_0.38_N. After ECE, a reflection peak appeared near 278 nm, and the peak reflectivity increased as the electrolyte was heated up from 25 to 65 °C, then decreased at 85 °C. Thus, the highest reflectivity 95% was obtained at 65 °C. To study the influence of electrolyte temperature on reflectivity, the cross-sectional morphology of DBRs prepared at different electrolyte temperatures was characterized by SEM. As shown in Fig. 1c–e, the nanopores showed an obvious layered distribution, concentrated in n-Al_0.62_Ga_0.38_N layers, while almost absent in u-Al_0.62_Ga_0.38_N layers due to doping selective etching. The effective refractive index (${n}_{eff}$) of n-Al_0.62_Ga_0.38_N layer with introduced air-filled nonapores can be described by the volume average theory^22^, ${n}_{eff}={\left[\varphi {n}_{air}^{2}+\left(1-\varphi \right){n}_{AlGaN}^{2}\right]}^{1/2}$, where φ is the porosity. The refractive index of Al_0.62_Ga_0.38_N in DUV region (λ = 278 nm) is about 2.58 leaving out the influence of doping^23^. As the electrolyte temperature increased, the nanopores size increased significantly, resulting in a larger refractive index contrast. This is the reason why the DBRs reflectivity increased with the temperature rising from 25 to 65 °C. The porosity of the n-Al_0.62_Ga_0.38_N layers etched at 65 °C was estimated to be 20% from the SEM image. Then, the ${n}_{eff}$ was calculated to be 2.35. The mismatch between simulation and experiment reflectivity is due to the finite nonuniformity of nanopores in n-Al_0.62_Ga_0.38_N layers and the scattering of DBRs surface. However, when the electrolyte temperature increased to 85 °C, the nanopores was too large and extend into the u-Al_0.62_Ga_0.38_N layer, as shown in Fig. 1f. Therefore, the periodic stacking of nanopores was destroyed, leading to the decrease of reflectivity compared with that at 65 °C. Subsequently, a 2-in. sample was etched at 25 V and 65 °C, and the reflectivity spectra were measured at the center, sub-center, and edge of the wafer, respectively, as shown in Fig. 2. The reflectivity at the three points was all over 95%, indicating that a wafer-scale DUV DBR was successfully prepared. The stopband of the reflectivity spectra was about 4.5 nm which was mainly limited by the short reflection wavelength and the low refractive index contrast. The peak wavelengths of the reflection spectra at the three points are 282 nm, 280.5 nm, and 279.5 nm, respectively, shifting slightly round 280 nm. The uneven doping and thickness can be the main cause of the slight shift.

In general, the ECE process of the anode has three stages: (i) Avalanche breakdown or Zener tunneling to generate holes; (ii) Anode oxidation of n-AlGaN at the interface between the film and the electrolyte; (iii) The oxides dissolving in the electrolyte to form nanopores^24^. The reaction can be expressed as:

1 $$2{Al}_{x}{Ga}_{1-x}N+{6OH}^{-}+{6h}^{+}\to {xAl}_{2}{O}_{3}+{\left(1-x\right)Ga}_{2}{O}_{3}+{3H}_{2}O+{N}_{2}$$

2 $${Al}_{2}{O}_{3}+{Ga}_{2}{O}_{3}+{12OH}^{-}\to {2AlO}_{3}^{-3}+{2GaO}_{3}^{-3}+{6H}_{2}O$$

**Figure 3:**
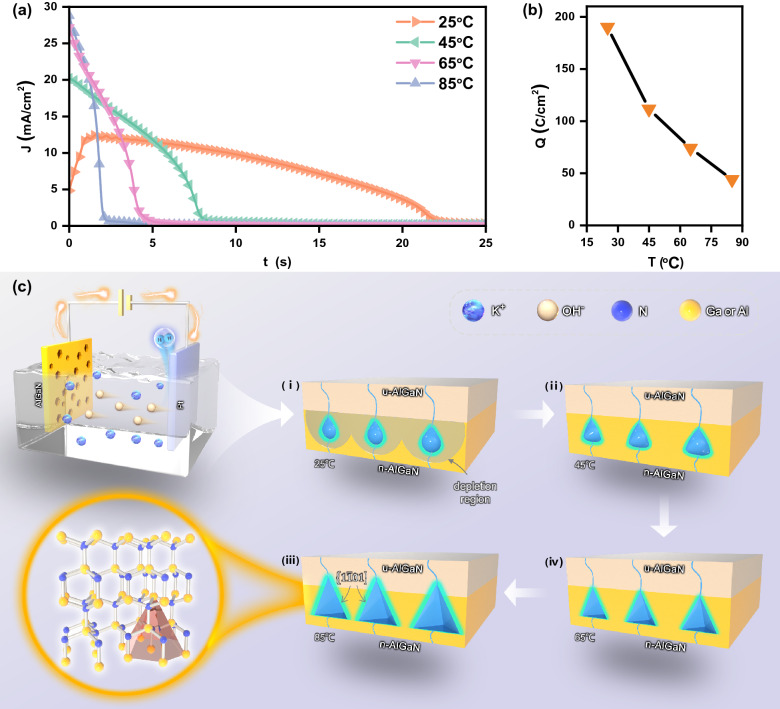
(**a**) J–t plots of DBRs etched in KOH solution at 25 V and different temperatures; (**b**) The integrated charge density as a function of temperature; (**c**) Schematic illustration of the electrochemical etching cell, nanopores etched in KOH solution at 25 V and different temperatures and AlGaN crystal structure.

Meanwhile, electrons (e^−^) are transferred to the Pt electrode, participating in the reduction reaction: ${2H}_{2}O+{2e}^{-}\to {H}_{2}+{2OH}^{-}$. According to Faraday’s law of electrolysis, the current density and the amount of charge passing through the source meter are proportional to the ECE rate and the mass of the AlGaN undergoing redox reaction, respectively. Figure 3a shows the J-t plots during ECE at 25 V. The current decreased to 0 after 23 s etching, indicating that the ECE process occurred within the first 23 s. When the temperature rose, due to the faster dissolution rate of oxides in the hotter KOH solution, the initial current density increased. Figure 3b shows the amount of charge consumed during ECE process, which was obtained by integrating J − t plots. The amount of charge decreased with the temperature rising, indicating that the mass of the AlGaN undergoing redox reaction decreased. Thus, it can be concluded that the increase of nanopores size with temperature is related to chemical etching other than ECE.

AlGaN film with III-polar plane was grown along the [0001], while that with N-polar plane grown along the opposite [000$\overline{1 }$]. Different from the III-polar plane, the N-polar plane is very easy to be etched in hot KOH solution^25^, and the etching rate is temperature and crystal face dependent^26,27^. The CE reaction can be expressed as:

3 $${Al}_{x}{Ga}_{1-x}N+{3OH}^{-}\to {xAlO}_{3}^{-3}+{(1-x)GaO}_{3}^{-3}+{NH}_{3}$$

Here we propose the formation process of nanopores under the combined action of ECE and CE. The ECE process makes n-Al_0.62_Ga_0.38_N layers porous and CE can be ignored at 25 °C, as shown in Fig. 3c_i_. The small nanopore size can be explained by the depletion model^28^. The thickness of space charge region (SCR) around nanopores is expressed as: ${d}_{SCR}=\sqrt{\frac{2\varepsilon {\varepsilon }_{0}{U}_{SCR}}{q{N}_{D}}}$, where *ε* is the dielectric constant, *ε*_*0*_ is the permittivity of AlGaN, *U*_*SCR*_ is the voltage drop across the depletion region, *q* is the charge of an electron, and *N*_*D*_ is the n-doping concentration. Due to the relatively low doping concentration of high Al composition AlGaN, the SCR around nanopores were thick. When the SCR overlapped, the space between nanopores was completely depleted, so the pore diameter cannot increase due to the lack of holes required for ECE. Moreover, ECE was isotropic without crystal plane selectivity, thus the nanopores are approximately circular. However, CE can no longer be ignored as the electrolyte temperature increases to 45 °C. The nanopores formed by ECE exposed the N-polar plane of the AlGaN film. Subsequently the CE of the N-polar plane takes place and the nanopores size increase, as shown in Fig. 3c_ii_. Undoubtedly, the effect of chemical etching is more obvious at 65 °C. The nanopores presents a triangular profile composed of {0001} and {1$\overline{1 }$01} family of planes, as shown in Fig. 3c_iii_, which is due to the relatively stable energy of the {1$\overline{1 }$01} family of planes^19,26^. Since CE has no silicon doping selectivity, when the solution temperature was 85 °C, the triangular nanopores could expand to u-Al_0.62_Ga_0.38_N layers and destroy the periodic stacking of the nanopores, as shown in Fig. 3c_iv_. This is the key reason why the reflectivity of DBR prepared in 85 °C KOH electrolyte decreases compared with that at 65 °C.

**Figure 4:**
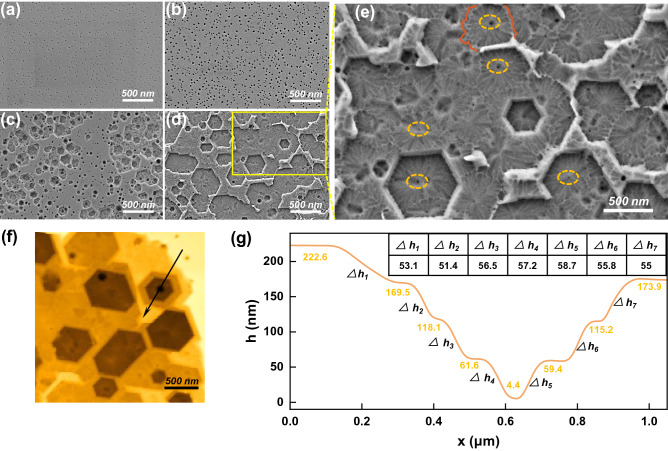
Surface SEM images of the DBR fabricated at 25 V in KOH solution at (**a**) 25 °C, (**b**) 45 °C, (**c**) 65 °C and (**d**) 85 °C; (**e**) Enlarged surface SEM image and (**f**) AFM of DBR fabricated at 25 V and 85 °C; (**g**) Depth profile of the hexagonal pit marked in (**f**).

The surface morphology of DBRs was also characterized, as shown in Fig. 4. The evenly distributed nanopores, about 35 nm in diameter, were formed on the DBR surface after etching at 25 V and 25 °C (Fig. 4a). As the temperature rose, the pore size increased at 45 °C (Fig. 4b), and the hexagonal pits appeared at 65 °C (Fig. 4c). When the temperature continued to rise to 85 °C, the surface was completely etched, which was ascribed to the coalescence of adjacent hexagonal pits (Fig. 4d). Due to the extremely low carrier concentration of the surface u-Al_0.62_Ga_0.38_N layer and the overlap of SCR, the nanopore size was small at 25 °C. However, in the depletion model, the SCR was not influenced by the electrolyte temperature, thus the formation of hexagonal pits cannot be attributed to ECE. Figure 4e is the enlarged image of the yellow box in Fig. 4d. It can be observed that the dendritic lines appeared around the nanopores. These lines were the paths for the diffusion and etching of KOH electrolyte in the n-Al_0.62_Ga_0.38_N layer. Due to the overlap of SCR, the dendritic lines around adjacent nanopores cannot be connected, thus forming a clear boundary, as marked by the red line. Therefore, it can be concluded that the nanopores acted as vertical downward channels for electrolyte in ECE process and the electrolyte spread laterally in n-Al_0.62_Ga_0.38_N layers^21,29^. In addition, the porous n-Al_0.62_Ga_0.38_N layer allowed the hot KOH solution to chemically etch the u-Al_0.62_Ga_0.38_N from the N-polar plane, which accelerated the etching of the surface u-Al_0.62_Ga_0.38_N layer. The hexagonal profile of the surface pits was formed due to the intersection of {0001} and {1 $\overline{1 }$ 01} family of planes. The surface morphology of the DBR etched at 85 °C and the depth of the hexagonal pits were also characterized and measured by AFM, as shown in Fig. 4f and g, respectively. The selected hexagonal pit exposed five step surfaces and seven step heights (∆h_x_). Except for Δh_1_ and Δh_2_, the other step heights are between 55 and 59 nm, consistent with the thickness (58 nm) of a pair of n-Al_0.62_Ga_0.38_N/u-Al_0.62_Ga_0.38_N (28 nm/30 nm). This confirms that a pair of n-Al_0.62_Ga_0.38_N/u-Al_0.62_Ga_0.38_N was etched as a whole where the n-Al_0.62_Ga_0.38_N layer was etched by both ECE and CE while the u-Al_0.62_Ga_0.38_N etched by CE only, thus forming hexagonal pits with uniform depth.

**Figure 5:**
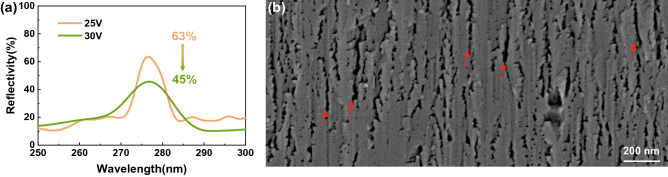
(**a**) The reflectivity spectra of DBRs prepared at 25 V or 30 V in KOH solution at 25 °C; (**b**) Cross-sectional SEM images of the DBRs etched at 30 V and 25 °C.

The influence of sample conductivity (doping) and anodization potential have been researched by Han et al.^28^. We have tried to play with the voltage for porosity tuning. However, the reflectivity of the DBR prepared at 30 V was lower than that at 25 V, as shown in Fig. 5. The relatively low Si doping concentration of n-Al_0.62_Ga_0.38_N (4 × 10^18^ cm^−2^) resulted in poor ECE selectivity at the higher voltage. Some vertical nanochannels (as marked by the red arrows) instead of periodically stacked nanopores were observed on the cross-sectional SEM images of the DBRs etched at 30 V. It was not feasible to achieve high reflectivity just by playing with the voltage.

## Conclusion

In conclusion, a wafer-scale DUV DBR was successfully fabricated by etching 40 pairs of n-Al_0.62_Ga_0.38_N/u-Al_0.62_Ga_0.38_N film in hot KOH aqueous solution. The EC expanded the size of nanopores formed by electrochemical etching, thereby enhancing the porosity of the n-AlGaN layers, and 95% reflectivity at 280 nm was achieved by optimizing the electrolyte temperature. The simple etching method can avoid various problems caused by lattice and thermal mismatch during epitaxial growth for preparing wafer-scale DUV DBR, which would be of significant importance for the fabrication of AlGaN based DUV microcavity devices.

## Data Availability

The data is available upon reasonable request to the corresponding author.
